# Normal sex differences in prenatal growth and abnormal prenatal growth retardation associated with 46,XY disorders of sex development are absent in newborns with congenital adrenal hyperplasia due to 21-hydroxylase deficiency

**DOI:** 10.1186/2042-6410-2-5

**Published:** 2011-05-05

**Authors:** Laura J Chalmers, Paul Doherty, Claude J Migeon, Kenneth C Copeland, Brianna C Bright, Amy B Wisniewski

**Affiliations:** 1 Department of Pediatrics, The University of Oklahoma College of Medicine-Tulsa, Tulsa, OK 74135, USA; 2 Department of Pediatrics, Division of Pediatric Endocrinology, The Johns Hopkins School of Medicine, Baltimore, MD 21287, USA; 3 Department of Pediatrics, Section of Diabetes and Endocrinology, The University of Oklahoma Health Sciences Center, Oklahoma City, OK 73117, USA

## Abstract

**Background:**

Congenital adrenal hyperplasia due to 21-hydroxylase deficiency is the most common presentation of a disorder of sex development (DSD) in genetic females. A report of prenatal growth retardation in cases of 46,XY DSD, coupled with observations of below-optimal final height in both males and females with congenital adrenal hyperplasia due to 21-hydroxylase deficiency, prompted us to investigate prenatal growth in the latter group. Additionally, because girls with congenital adrenal hyperplasia are exposed to increased levels of androgens in the absence of a male sex-chromosome complement, the presence or absence of typical sex differences in growth of newborns would support or refute a hormonal explanation for these differences.

**Methods:**

In total, 105 newborns with congenital adrenal hyperplasia were identified in our database. Gestational age (weeks), birth weight (kg), birth length (cm) and parental heights (cm) were obtained. Mid-parental height was considered in the analyses.

**Results:**

Mean birth weight percentile for congenital adrenal hyperplasia was 49.26%, indicating no evidence of a difference in birth weight from the expected standard population median of 50th percentile (*P *> 0.05). The expected sex difference in favor of heavier males was not seen (*P *> 0.05). Of the 105 subjects, 44 (27%; 34 females, 10 males) had birth length and gestational age recorded in their medical chart. Mean birth length for this subgroup was 50.90 cm (63rd percentile), which differed from the expected standard population median of 50th percentile (*P *= 0.0082). The expected sex difference in favor of longer males was also not seen (*P *> 0.05).

**Conclusion:**

The prenatal growth retardation patterns reported in cases of 46,XY disorders of sex development do not generalize to people with congenital adrenal hyperplasia due to 21-hydroxylase deficiency. Sex differences in body weight and length typically seen in young infants were not seen in the subjects who participated in this study. We speculate that these differences were ameliorated in this study because of increased levels of prenatal androgens experienced by the females infants.

## Background

Several reports have suggested an association between low birth weight and atypical development of the male reproductive system. For example, low birth weight is more common in newborns with isolated hypospadias [1-9] and in genetic males with a disorder of sex development (DSD) [10], particularly when the DSD is idiopathic in nature [11-14]. Birth weight and length in humans are sexually dimorphic traits; healthy male newborns weigh more and are longer than their female counterparts [7,15]. Male newborns also have a greater head circumference and less body fat than females, as indicated by decreased skin-fold thickness and whole-body plethysmography [16]. Together, these observations point to the possibility that lower levels of androgen production or action *in utero *that would be expected for a male contributes not only to undermasculinization of the male reproductive system, but also to decreased anthropometric measures in newborns [7].

Low birth weight is of concern, independently of its relationship with atypical reproductive development, because of the association with development of metabolic, endocrine and cardiovascular diseases later in life [17]. Furthermore, some newborns who are born small fail to achieve catch-up growth as they develop.

Congenital adrenal hyperplasia (CAH) due to 21-hydroxylase deficiency (21-OH) is the commonest cause of DSD in 46,XX individuals. Females with this condition are exposed during early development to levels of androgens that can be similar to the atypically low levels of androgens experienced by some genetic males with DSD. Furthermore, both under- and over-treatment of CAH with glucocorticoids results in a final adult height that is shorter than that predicted from mean parental height (MPH) [18]. A better understanding of crucial periods of growth, including the prenatal period, should prove beneficial in efforts to optimize final heights in people with CAH [19].

To our knowledge, only three published studies have analyzed prenatal growth in newborns with CAH due to 21-hydroxylase (21-OH) deficiency. The first study reported normal birth weight [20], and the second reported an increase in birth weight in affected girls but not boys [21]. These data indicate that, unlike cases of 46,XY DSD, 'small for gestational age' cannot be generalized to the most common presentation of 46,XX DSD. However, neither study controlled for the possible confounding effects of parental height on birth weight. The third study measured linear growth in boys and girls with classic CAH during the first 3 years of life. and reported that birth length was above average for affected children; however, neither sex differences nor body mass data were considered in thst study [22].

The goal of the present study was to retrospectively evaluate birth weight and length in newborns with CAH due to 21-OH deficiency whereas controlling for the influence of parental height. Furthermore, because female newborns with CAH due to 21-OH deficiency are exposed to increased levels of androgens before treatment in the absence of a male sex-chromosome complement, their growth patterns allow us to discriminate between hormonal versus genetic influences in the context of sexually dimorphic anthropometric measures.

## Methods

The study was approved by the institutional review boards of the University of Oklahoma Health Sciences Center and the Johns Hopkins School of Medicine, informed consent was not required.

A retrospective chart review was performed on all pediatric endocrine charts from both clinics collected over the past 50 years. Subjects with CAH due to 21-hydroxylase deficiency were identified from each institution and reviewed (105 in total). Gestational age (weeks), birth weight (kg), birth length (cm) and parental heights (cm) were obtained when documented in the chart. Subjects of gestational age < 37 weeks or > 44 weeks were omitted from the analyses. A single instance of twin gestation was identified in our data, but was excluded from the current analyses based on delivery at 34 weeks gestational age.

Birth weight and length information were entered into a software program (Epi Info™. version 3.4.1; 1. Epi Info™; http://www.cdc.gov/epiinfo), yielding birth weight, length, and weight, for, height percentiles and z-scores. MPH was calculated based on the formula: (maternal height (cm) ± paternal height (cm) ± 13 cm (+13 cm for males and -13 cm for females) [23].

Wilcoxon signed-rank tests were used to test whether birth weight and length percentiles differed from the standard median (50th percentile), and the Mann-Whitney *U*-test and Student *t*-test were used to examine the difference in study variables between male and female subjects with CAH. Spearman correlation coefficients were also calculated to determine the strength of the relationship between MPH and both birth weight (kg) and birth length (cm).

## Results

Of the 105 subjects, 91% were born between 37 and 40 weeks. Gestational age ranged from 37 to 43 weeks (mean ± SD 39.91 ± 1.04). Mean birth weight for CAH subjects was 3.42 kg, with an average female and male weight of 3.39 and 3.50 kg, respectively (Table 1). No sex difference in weight was seen (*t *_(103) _= -1.18, *P *> 0.05). Mean birth weight percentile for CAH was 49.26%, indicating no difference in birth weight from the expected standard population median of 50th percentile (*S *= -117, *P *> 0.05). There was no sex difference in birth weight percentile between male and females subjects (*U *= 1453, *P *> 0.05).

**Table 1:** Birth weight and z-scores for subjects with congenital adrenal hyperplasiadue to 21-OH deficiency.

	Female (n = 78) (mean ± SD)	Male (n = 27) (mean ± SD)	Total (n = 105) (mean ± SD)
Birth weight^1 ^(kg)	3.39 ± 0.47	3.50 ± 0.41	3.42 ± 0.45

z-score	0.01 ± 0.97	-0.01 ± 0.77	0.00 ± 0.92

^1^Mean birth weight percentile did not differ from the 50th percentile (*P *> 0.05) and no sex difference was seen (*P *> 0.05).

Of the 105 subjects with CAH, 44 (42%; 34 females and 10 males) had birth length and gestational age recorded in their chart (Table 2). Mean birth length for the group of subjects was 50.90 cm, with average female and male length of 50.79 and 51.30 cm, respectively. There was no sex difference in birth length (*U *= 237, *P *> 0.05). The mean birth length percentile for all subjects was 63.11%, which differed from the expected standard population median of the 50th percentile (*S *= 221, *P *= 0.0082). In total, 68 subjects had their MPH calculated, but only 30 subjects had four data points available (gestational age, birth weight and length and MPH) to analyze. There was no significant correlation between birth weight (*r *= 0.13, *P *> 0.05) or length (*r *= 0.15, *P *> 0.05) percentiles and MPH.

**Table 2:** Birth length and z-scores for subjects with congenital adrenal hyperplasia due to 21-OH deficiency.

Parameter	Results, mean ± SD		
	
	Female (n = 34)	Male (n = 10)	Total (n = 44)
Birth length (cm)^1^	50.79 ± 2.65	51.30 ± 3.64	50.90 ± 2.86
z-Score	0.53 ± 0.99	0.51 ± 1.39	0.53 ± 1.07

^1^Mean birth length percentile exceeded the population 50th percentile (*P *= 0.0082) and no sex difference was seen (*P *> 0.05).

## Discussion

The sex ratio at birth in CAH, an autosomal recessive disorder, is almost equal [24]. Female newborns are detected more readily at birth because ambiguity of their external genitalia resulting from excess androgen exposure *in utero *[25]. Before the advent of newborn screening for CAH due to 21-OH deficiency, male newborns with CAH had delayed or missed diagnoses, resulting in salt-wasting adrenal crises and increased mortality rates [26-29]. Thus, the 2.5:1 female:male ratio for CAH in our data reflects the retrospective nature of the cases that presented prior to the 1970s.

Consistent with results from previous investigations [20], infants in the current study had birth weights in the expected population distribution median; however, birth length percentile was higher than the expected population distribution median [22]. Measuring birth length can be difficult. The neonatal cranial molding necessary during delivery, timing after delivery, and flexion of the infant's body are a;; factors that contribute to this difficulty [30]. However, studies report intra- and inter-examiner reliability of birth length measurement of >1.0 cm [31,32]. This exceeds the 7 mm intra- and inter-examiner reliability allowed during the standardization process for the World Health Organization Child Growth Standard curves [33]. Furthermore, our results are consistent with those of Bonfig *et al*. [22], who reported results from a prospective study of 51 patients. For all these reasons, we conclude that unlike the retarded growth pattern reported in cases of idiopathic DSD in genetic males, birth weight and length are not inhibited by CAH due to 21-OH deficiency.

In addition to increased birth length seen in the study cohort overall, we failed to detect the expected sex difference in favor of birth length for male infants in our investigation. We speculate that this is due to increased linear growth of both females and males with classic CAH resulting from increased levels of adrenal androgens before birth. Androgens were increased for all newborns in this cohort, as no mothers received dexamethasone treatment during pregnancy and all females had ambiguous genitalia at birth.

Previous work by our group has shown that sex differences in body mass and composition in infants disappear by 6 months of age, when the testes of healthy males stop producing androgens. Further support for our proposition that early androgen exposure drives sex differences, at least in part, is our observation that newborns with 46,XY DSD due to complete androgen insensitivity syndrome have birth weights that do not differ from unaffected female newborns [34]. Taken together, these observations strongly suggest that prenatal androgen exposure, rather than the possession of a male chromosome complement, stimulates linear growth and body composition in humans in a male-typical manner.

Athough evidence from multiple sources points to the role of androgens in prenatal growth, androgens are not alone sufficient for normal intrauterine growth. Several other factors are required, including the insulin-like growth factor (IGF) system, adequate nutrient and oxygen supply to the fetus, and normal imprinting of genes involved in early growth. For example, children with IGF-1 deficiency have depressed patterns of prenatal growth despite their exposure to typical levels of androgens during gestation [35]. Additionally, boys with Russell-Silver syndrome experience intrauterine growth retardation and hypospadias despite normal early androgen production [36].

For newborns with CAH due to 21-OH deficiency, increased birth length could be due to a relative deficiency of cortisol, which has well-known growth-suppressing effects, or to excess levels of adrenal hormone precursors *in utero*, including 17-hydroxyprogesterone. Studies of non-human animal models indicate that maternal activation of the hypothalamic-pituitary-adrenal axis during pregnancy results in alterations in offspring body mass, but not skeletal growth [37-39]. Conversely, non-human animals hypophysectomized *in utero *have reduced body mass [40], limb length [41,42] and crown rump length [42], the opposite outcome of the increased birth length seen in the current study.

It seems that newborns with CAH have already experienced some alterations in linear growth compared with their unaffected counterparts. Possibly, some of the difficulty seen in effecting normal linear growth throughout childhood of individuals with this disorder may be independent of post-natal cortisol-replacement regimens.

## Conclusion

The prenatal growth retardation patterns reported in cases of 46,XY DSD do not generalize to individuals with CAH due to 21-OH deficiency. Sex differences in body weight and length typically seen in young infants were not observed in the subjects who participated in this study. We speculate that these differences were ameliorated in this study due to increased levels of prenatal androgens experienced by the female infants.

## Competing interests

The authors declare that they have no competing interests.

## Authors' contributions

The work presented here was carried out in collaboration between all the authors. All authors read and approved the final manuscript.
